# Transurethral Resection of Prostate Abscess: Is It Different from Conventional Transurethral Resection for Benign Prostatic Hyperplasia?

**DOI:** 10.1155/2013/109505

**Published:** 2013-06-11

**Authors:** Neeraj Kumar Goyal, Apul Goel, Satyanarayan Sankhwar, Divakar Dalela

**Affiliations:** Department of Urology, King George's Medical University, Lucknow 226003, India

## Abstract

*Purpose*. To present our experience of prostate abscess management by modified transurethral resection (TUR) technique. *Methods*. Seventeen men with prostate abscess undergoing TUR between 2003 and 2011 were retrospectively analyzed. Details of demography, surgical procedures, complications, and followup were noted. *Results*. With a mean age of 61.53 ± 8.58 years, all patients had multifocal abscess cavities. Initially, 6 men underwent classical TUR similar to the technique used for benign prostatic enlargement (group 1). Next, 11 men underwent modified TUR (group 2) in which bladder neck and anterior zone were not resected. The abscess cavities resolved completely, and no patient required a second intervention. One patient in group 1 and three in group 2 had postoperative fever requiring parenteral antibiotics (*P* = 0.916). Three patients in group 1 had transient urinary incontinence, whereas none of the patients in group 2 had this complication (*P* = 0.055). Four and five men in group 1 and 2 reported retrograde ejaculation, respectively (*P* = 0.740). *Conclusion*. The modified technique of prostate resection edges over conventional TURP in the form of reduced morbidity but maintains its high success rate for complete abscess drainage. It alleviates the need for secondary procedures, having an apparent advantage over limited drainage techniques. Use of this technique is emphasized in cases associated with BPH and lack of proper preoperative imaging.

## 1. Introduction

In the modern antibiotic era, prostatic abscess is a rarely encountered entity, particularly in developed countries [1, 2]. However, in developing countries it continues to be a significant health problem and can result in severe complications and even death on account of delayed diagnosis or inadequate management [3]. Therapeutically, it requires some form of surgical intervention as a medical treatment as monotherapy is usually not sufficient [3]. The management options include transrectal ultrasound- (TRUS-) guided aspiration/tube drainage, transurethral incision over abscess (TUI), transurethral deroofing of the abscess cavity (TUD), or formal transurethral resection of prostate (TURP) [3–9]. No management algorithm is currently available to guide the surgical drainage, and the decision is usually based on the preference of the treating physician. Although TUD and TURP have been described as two separate procedures for prostatic abscess, the techniques are overlapping and poorly defined in the contemporary literature. Similarly, the indications of a particular procedure (whether deroofing or resection) and complications are not well documented. A retrospective analysis of data of men with prostate abscess treated by transurethral resection was done to document the technique of the procedure and its complications.

## 2. Material and Methods

Data of all the patients diagnosed with prostatic abscess and managed with TURP between July 2003 and January 2011 was analyzed retrospectively. Patients managed by other means were excluded from the present study. Studied parameters included the age of the patients, clinical presentation, risk factors, radiological findings, treatment details, and the complications encountered. The details of surgical resection and all the followup visits were recorded. The complications were reported according to the modified Clavien classification system [10]. All patients had a repeat abdominal or transrectal ultrasonography at first followup visit to look for any residual abscess. After that, the followup visits were every six months for 1 year and annually thereafter. Men with incomplete data were excluded from the study.

## 3. Statistical Analysis

Data were summarized as mean ± SD and percentage. Groups were compared by independent Student's *t*-test, chi square (*χ* ^2^) test, and proportion *z*-test wherever applicable. A two-sided (*α* = 2)*P* < 0.05 was considered statistically significant. 

## 4. Results

**Figure 1:**
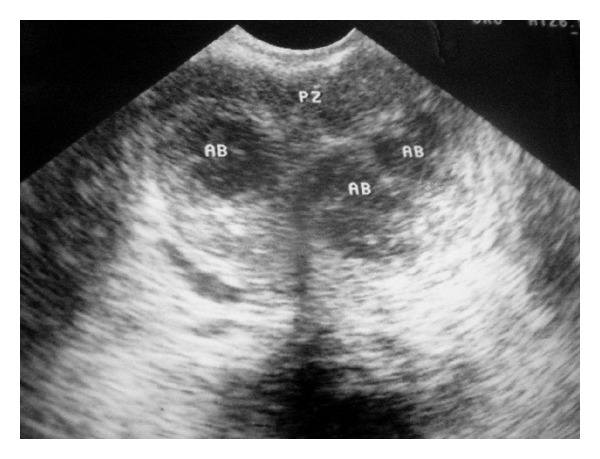
TRUS image of a patient showing multiple prostatic abscesses (AB) involving both lobes with relative sparing of the peripheral zone (PZ).

**Figure 2:**
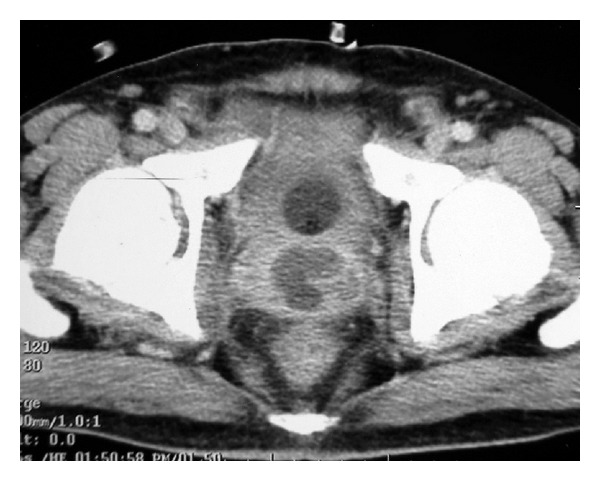
Contrast-enhanced CT image of a patient showing multiloculated prostatic abscesses involving almost whole of the prostate. Patient with prior failed TRUS-guided aspirationgo responded well after TURP.

Forty-four men were treated for prostatic abscess during this period. Seventeen, out of them, fulfilled the selection criteria and were included in the present study. The mean age was 61.53 ± 8.58 years (range 43 to 72). The mode of presentation was dysuria and lower urinary tract symptoms in 14, recurrent fever in 9, and urinary retention in 7 patients. Seven men with history of urinary retention were on indwelling urethral catheter. Risk factors included diabetes mellitus in 9, recurrent urinary retention with history of urethral catheterization in 5, and prostatic biopsy in two, which were not mutually exclusive. There was no documented risk factor in 5 patients. Fluctuation on digital rectal examination was present in 6 patients only. Abdominal and/or TRUS confirmed the presence of abscess in all but one (Figure 1). Seven patients had undergone TRUS, while two men underwent contrast-enhanced CT scan in their preoperative evaluation (Figure 2). In one patient, abscess was diagnosed intraoperatively, where multiple small abscesses were found throughout the gland. In three men, TURP was done as a secondary procedure after failed TRUS-guided aspiration. All patients were receiving parenteral antibiotics at the time of intervention. Intraoperatively, the abscess cavities were localized to be centrally located in eight patients, more in the peripheral region in seven, and total prostatic involvement in two men. All the patients had multiloculated or multifocal abscess cavities. All procedures were performed under spinal anesthesia. The initial six men had undergone the classical transurethral resection of prostate similar to the technique performed for benign prostatic enlargement (group 1). Later on, the other 11 men underwent transurethral resection as a modified procedure (group 2). In the modified resection group, the bladder neck was not resected, and no resection was done between 10 and 2 o'clock at the roof. Rest of the procedure was completed as standard TURP. The abscesses resolved completely in all the patients. Catheter-free trial was given after a mean interval of 5.7 days (range 3 to 10 days) with all men voiding successfully. The perioperative and postoperative details of both groups are summarized in Table 1. Four patients continued to have fever (Clavien grade ll) postoperatively, which responded to culture-specific antibiotics. Three men in group 1 (50%) reported stress urinary incontinence (Clavien grade l) early in the postoperative period, whereas no patient in group 2 had any continence-related problem (*P* = 0.055). The transient stress incontinence reported in group 1, subsided at 3 weeks in one patient, and improved totally with conservative management at 3 months in rest of the two patients. Retrograde ejaculation was reported by four patients in group 1 and five men in group 2 (*P* = 0.74). The mean duration of followup was 58 months (range 6–92 months). All patients on followup are asymptomatic, and none require=ed subsequent medical or surgical intervention.

## 5. Discussion

The technique of transurethral resection for benign prostatic hyperplasia is well defined and standardized. However, the technique of transurethral drainage of prostatic abscess is not clear. The available options include limited interventional techniques (TUI and TUD) [5, 6, 8, 9] and the “more” invasive method like TURP [1, 8, 9]. Although all these methods have been described to be effective for draining prostate abscess, frequent complications encountered include septicemia, hemorrhage, residual abscess, retrograde ejaculation, and urinary incontinence. The techniques of TUI and TUD have the advantages of minimal invasion with a disease-specific treatment approach and less chances of complications [3, 6]. However, the major disadvantage of these methods is the risk of incomplete drainage of abscess. The limited drainage techniques may be adequate in a patient with single large abscess but in multifocal or multiloculated abscess cavities, and these are not sufficient [11]. To overcome the problem of incomplete drainage, Kinahan et al. described the use of intraoperative TRUS guidance to ensure complete transurethral drainage of prostatic abscess in a patient [11]. Although this technique sounds practical, it involves additional trained manpower and extra equipment and is cumbersome to perform. Secondly, many elderly men with prolonged history of lower urinary tract symptoms (LUTS) have a component of benign prostatic hyperplasia (BPH), which may not respond to limited drainage or may require another surgical intervention (in the form of TURP) in the lifetime [3, 12].

The diagnosis and localization of prostatic abscess have been facilitated with the advent of TRUS and axial imaging [4–6, 13, 14]. These modalities can exactly define the location, size, and number of abscess cavities and help in the management. However, performing TRUS may not be feasible in all the men as it is highly painful in the presence of prostatic abscess, and CT scan is usually not indicated to localize prostatic abscess [9]. Transabdominal USG, although sufficient to make a diagnosis, usually does not delineate the exact anatomical details of prostatic abscess. Unifocal or multifocal nature of the abscess cannot be ascertained clinically. Therefore, while performing TUI or TUD in an inadequately imaged patient, an inherent chance of incomplete drainage is always there.

Many authors have advocated classical TURP for treating prostatic abscess, either because of incomplete response to limited drainage procedures or as a primary procedure because of associated BPH. In major published studies, up to one third of the patients suffering from prostatic abscess ultimately required TURP [8, 9, 12]. In one of the largest series of 25-patients by Dajani and O'Flynn, two patients underwent TURP as the primary procedure because of associated prostate enlargement. Four required complete TURP before hospital discharge, and three more patients underwent TURP at a later date because of persistent symptoms. So, ultimately nine patients (36%) required complete prostate resection for abscess [12]. Similarly, in other series of 48 patients by Bhagat et al. [8], 14 patients underwent complete TURP because of associated BPH symptoms and prostatic enlargement, while 17 men underwent limited transurethral abscess drainage. In a series of 18 patients reported by Ludwig et al. [9], transurethral deroofing was done in 3 patients when the abscess was located just adjacent to the prostatic urethra, but one of the three required a repeat resection for elimination of infection. In an MRSA-caused prostatic abscess, reported by Park et al. [15], patient did not improve with TRUS-guided abscess drainage because of high viscosity pus and required TURP the very next day. His worsening clinical condition improved following TURP. Aravantinos et al. [3] treated prostatic abscess in 7 patients with trans-rectal placement of drainage tube. However, two patients required TURP later on as a selective procedure for persistent bladder outlet obstruction.

At our centre, we have been performing TURP for prostatic abscess for the last many years. One reason was that in many of our patients, adequate preoperative imaging had not been available. However, with evolution of percutaneous techniques and general acceptance of TUD in the urologic community, we changed our technique and started the modified approach. We felt that our modified method of drainage circumvented the limitations of incomplete drainage associated with TUI or TUD and would be associated with lesser complications as those associated with TURP (hemorrhage, retrograde ejaculation, and incontinence). Attention was paid not to perforate the venous sinuses to avoid the risks of septicemia and hemorrhage. To document the advantages and complications of our modified approach we retrospectively analyzed our data.

We found that classical TURP for prostatic abscess was associated with a high incidence (3 out of 6 men, i.e., 50%) of transient urinary incontinence (Clavien grade I). Although the exact reason for this observation is not well understood, a tendency to overresect so as to drain all the abscess cavities was probably the major cause. Secondly, although not documented, an inflammatory reaction in the region of external sphincter may be responsible for transient sphincter dysfunction. Another possible reason could be bladder overactivity in response to inflammation of the prostatic fossa. The incontinence seen in our patients was temporary, and all men recovered with conservative management within 3 months. This high rate of incontinence in the present study has not been previously reported by others [8, 12]. This may be because of paucity of contemporary data on classical TURP (for prostatic abscess) and underreporting of this complication as it is usually temporary. In our study, men who underwent modified resection (group 2) did not encounter any continence-related problem (*P* = 0.055). Bladder neck sparing with limited anterior resection is the possible reason for this advantage over conventional resection. Postoperative urinary incontinence although transient is a dreadful complication causing anxiety both to the patient and the operating surgeon. Retrograde ejaculation (Clavien grade ll) was noted in both groups and was more common in group 1, although statistically not significant (*P* = 0.740). All the patients responded well to the resection, and the abscess cavities resolved completely in all of them. Four of the patients had fever (Clavien grade II) postoperatively (one out of six in group 1 and three out of eleven in group 2), which responded to culture-specific parenteral antibiotics. But no patient had features of sepsis. No patient had excessive blood loss requiring blood transfusion in either group. 

There is paucity of the recent literature on transurethral drainage of prostatic abscess. The reasons are the falling incidence in the developed countries and most cases being managed successfully with parenteral antibiotics and TRUS-guided aspiration. As no standard technique is described in the literature regarding drainage of prostate abscess, we recommend our modified resection technique in patients requiring transurethral drainage of prostatic abscess, especially if adequate imaging is lacking. An additional advantage of our modified approach is that complete resection of the infected glandular prostate tissue is ensured. We also noticed that the anterior zone of fibromuscular stroma is not involved in prostatic abscess further justifying the modified method. The mean age of patients in our study was 61.53 ± 8.58 years, and number of young patients was very low. In younger patients, the proper preoperative evaluation is mandatory, and other minimally invasive treatment measures should be preferred over TURP in view of high risk of retrograde ejaculation after complete resection.

Our study has certain limitations, the most important being comparison of this technique with standard TURP for BPH. TURP is rarely performed for managing abscess these days, as the majority of the prostate abscesses are being managed successfully using minimally invasive modalities. Other limitations include the small number of cases, retrospective nature of the study, and short followup data. Though the difference between two groups was not statistically significant due to the small sample size, the incidence of postoperative incontinence was seen just approaching the statistical significance (*P* = 0.055). Due to these limitations, we cannot recommend it as a “wholesale” procedure in all the situations but it definitely appears to have a place in the management of patients requiring more liberal drainage.

## 6. Conclusions

The modified resection technique ensures complete drainage of all the abscess cavities and avoids the need of any secondary procedure on long-term followup. It has no extra morbidity, compared to other limited resection procedures, and can be recommended for treating prostatic abscess whenever the situation demands, particularly when proper preoperative imaging is not available or when associated with a component of BPH.

## Figures and Tables

**Table 1:** Demographics characteristics and treatment-related side effects of two groups.

Parameters	Group 1 (classical resection) (*N* = 6)	Group 2 (modified resection) (*N* = 11)	*P* value
Age (yrs): mean ± SD (range)	60.83 ± 9.32 (47–72)	61.90 ± 8.59 (43–70)	0.815
Operating time (min): mean ± SD (range)	54.16 ± 12.19 (38–70)	55.00 ± 12.01 (42–80)	0.893
Abscess localization			
Central	3 (50.0%)	5 (45.4%)	0.526
Peripheral	3 (50.0%)	4 (36.4%)	
Pan-prostatic	0 (0.0%)	2 (18.2%)	
Diabetes mellitus	2 (33.3%)	7 (63.6%)	0.491
Blood transfusion	0 (0.0%)	0 (0.0%)	1.000
Residual abscess	0 (0.0%)	0 (0.0%)	1.000
Temporary incontinence (Clavien grade I)	3 (50.0%)	0 (0.0%)	0.055
Postoperative fever (Clavien grade II)	1 (16.7%)	3 (27.3%)	0.916
Retrograde ejaculation (Clavien grade II)	4 (66.7%)	5 (45.4%)	0.740

## References

[1] Jacobsen JD, Kvist E (1993). Prostatic abscess: a review of literature and a presentation of 5 cases. *Scandinavian Journal of Urology and Nephrology*.

[2] Weinberger M, Cytron S, Servadio C, Block C, Rosenfeld JB, Pitlik SD (1988). Prostatic abscess in the antibiotic era. *Reviews of Infectious Diseases*.

[3] Aravantinos E, Kalogeras N, Zygoulakis N, Kakkas G, Anagnostou T, Melekos M (2008). Ultrasound-guided transrectal placement of a drainage tube as therapeutic management of patients with prostatic abscess. *Journal of Endourology*.

[4] Cytron S, Weinberger M, Pitlik SD, Servadio C (1988). Value of transrectal ultrasonography for diagnosis and treatment of prostatic abscess. *Urology*.

[5] Granados EA, Riley G, Salvador J, Vicente J, Krieger JN, Uehling DT (1992). Prostatic abscess: diagnosis and treatment. *Journal of Urology*.

[6] Bansal P, Gupta A, Mongha R Minimally-invasive management of prostatic abscess: the role of transrectal ultrasound. *Urology Annals*.

[7] Basiri A, Javaherforooshzadeh A (2010). Percutaneous drainage for treatment of prostate abscess. *Urology Journal*.

[8] Bhagat SK, Kekre NS, Gopalakrishnan G, Balaji V, Mathews MS (2008). Changing profile of prostatic abscess. *International Brazilian Journal of Urology*.

[9] Ludwig M, Schroeder-Printzen I, Schiefer HG, Weidner W (1999). Diagnosis and therapeutic management of 18 patients with prostatic abscess. *Urology*.

[10] Dindo D, Demartines N, Clavien P-A (2004). Classification of surgical complications: a new proposal with evaluation in a cohort of 6336 patients and results of a survey. *Annals of Surgery*.

[11] Kinahan TJ, Goldenberg SL, Ajzen SA, Cooperberg PL, English RA (1991). Transurethral resection of prostatic abscess under sonographic guidance. *Urology*.

[12] Dajani AM, O’Flynn JD (1968). Prostatic abscess: a report of 25 cases. *British Journal of Urology*.

[13] Thornhill BA, Morehouse HT, Coleman P, Hoffman-Tretin JC (1987). Prostatic abscess: CT and sonographic findings. *The American Journal of Roentgenology*.

[14] Barozzi L, Pavlica P, Menchi I, De Matteis M, Canepari M (1998). Prostatic abscess: diagnosis and treatment. *The American Journal of Roentgenology*.

[15] Park SC, Lee JW, Rim JS (2011). Prostatic abscess caused by community-acquired methicillin-resistant Staphylococcus aureus. *International Journal of Urology*.

